# Difference in drug cost between private and public drug plans in Quebec, Canada

**DOI:** 10.1186/s12913-022-07611-4

**Published:** 2022-02-14

**Authors:** Chamoun M., Forget A., Chabot I., Schnitzer M., Blais L.

**Affiliations:** 1 Faculty of Pharmacy, Université de Montréal, Montreal, QC Canada; 2 Centre Intégré Universitaire de Santé Et de Services, sociaux du Nord-de-l’île-de-Montréal, Montreal, QC Canada; 3 Endowment Pharmaceutical Chair AstraZeneca in Respiratory Health, Montreal, QC Canada

**Keywords:** Drug insurance, Drug plan, Drug cost difference

## Abstract

**Background:**

We expect a difference in drug cost between private drug plans and the Public Drug Plan (PDP) because the dispensing fee is fixed and regulated by the PDP for publicly insured patients, whereas it is determined freely by the pharmacy owner for privately insured patients. This study compared the drug cost of Quebec residents covered by private drug plans with those covered by PDP.

**Methods:**

We used a sample of prescriptions filled between 1 January 2015 and 23 May 2019 selected from reMed, a database of Quebecers’ drug claims. We created strata of prescriptions filled by privately insured patients and matched them with strata of prescriptions filled by publicly insured patients based on the Drug Identification Number, quantity dispensed, number of days of supply, pharmacy identifier, and a date corresponding to the publication of List of Medications of *Régie de l’Assurance Maladie du Québec*. The differences in drug cost between private plans and the PDP were analyzed with linear regression models using prescription strata as the unit of analysis.

**Results:**

Based on 38 896 prescription strata, we observed that privately insured patients payed $9·35 (95% confidence interval [CI]: 5·58; 13·01) more on average per drug prescription than publicly insured patients, representing a difference of 17·6%.

**Conclusions:**

This study showed that, on average, drug cost is substantially higher for privately insured Quebecers. Knowing that adherence to treatment is affected by drug cost, these results will help public health authorities to make informed decisions about drug policies.

## Introduction

Since 1997, every Quebec resident has been required to have prescription drug insurance coverage at all times. There are two types of insurance plan: the Public Drug Plan (PDP), which is administered by the *Régie de l’Assurance Maladie du Québec* (RAMQ), and private plans (group insurance or employee benefits plans) [1]. Individuals and their family eligible for a private plan must join that plan. As of 2018, approximately 45% of Quebecers were not eligible for private coverage, were older than 65 years, or had access to Social Assistance and Social Solidarity, and thus where covered by the PDP [2]. Direct patient contributions to payment of the covered drugs they purchase vary by type of drug plan; i.e., patients covered by the PDP pay a monthly deductible of $21·75 plus 37·0% of the cost of each prescription filled as copayment, up to a maximum monthly contribution of $93·08 ($1 117 per year). After reaching the maximum contribution, covered drugs are free of charge until the end of the month. Privately insured patients pay a yearly deductible ranging from $0 to $100 and a copayment of 0% to 37%, with a maximum yearly contribution of $1 117 [3, 4]. After reaching the maximum contribution, the covered drugs are free of charge until the end of the year.

Drug cost in Quebec has three components: ingredient list price, wholesaler mark-up, and dispensing fee. The first two components are regulated by the RAMQ and are the same for patients covered by the PDP or by private drug plans. The third component is fixed by the RAMQ (between $8·50 and $9·49) for beneficiaries of the PDP. For patients covered by private drug plans, the dispensing fee is not regulated and is established by pharmacy owners to promote business profitability. Therefore, the drug cost is the same in all pharmacies for patients covered by the PDP, but may vary among pharmacies for patients covered by a private drug plan [1, 5].

Drug cost has been identified as a barrier to adherence to prescribed treatment [6]. Patients who spend more than USD 100 per month for drugs are 5·57 times more likely to be non-adherent than patients who spend less than USD 50 per month (*p* < 0·001) [7]. Low adherence is associated with lower treatment efficacy, disease complications, and increase in health care expenditure [6]. In the United States and Canada, non-adherence is estimated to cost annually to the healthcare systems USD 100 billion and CAD 4 billion, respectively [8, 9].

According to the studies conducted in the United States and Canada, including the province of Quebec, the average drug cost is between 13 and 70% higher for privately insured patients than publicly insured patients [10–18]. However, the methodological limitations of previous studies and reports prevent us from drawing reliable conclusions from the reported wide range of differences in drug cost. These limitations include small sample size, the analysis of only one drug or one class of drugs, and failure to assess whether the observed differences are statistically significant or whether they are due to hazard. Finally, the previous studies compared patients with private and public drug insurance who had purchased different drugs with different quantities dispensed, formulations, and numbers of days supplied, and so may have reported confounded cost differences.

The overarching aim of the study is to provide a portrait of drug costs for Quebec residents covered by private and public drug plans to patients, healthcare professionals and decision makers. The primary objective was to estimate the average difference in drug cost between patients covered by private drug plans and the PDP in Quebec. We used provincial electronic prescription records and a design that minimizes confounding by directly contrasting the purchase of identical drugs between private dug plans and the PDP. The secondary objective was to estimate the average difference in drug cost separately for generic and innovator (or brand name) drugs between patients covered by private drug plans and those covered by the PDP.

## Methods

### Source of data

This study was performed using the *Registre de données sur les médicaments* (reMed) database and all methods were performed in accordance with the relevant guidelines, regulations and approved by an appropriate ethics committee. This database includes longitudinal patient-level claims data for prescriptions filled at community pharmacies by a sample of Quebec residents enrolled in community pharmacies, medical clinics, or blood test facilities from different regions of Quebec since 2008. At enrollment, participants were covered by a private drug plan and had to be age < 65 years. Patients were kept in the database if they switched to the PDP or reached the age of 65 years. The data, including drug name, formulation, dose, quantity dispensed, Drug Identification Number (DIN; a unique identifier of all drug products sold in a specific dosage form in Canada), date of prescription being filled, number of days supplied, drug insurance type (private or PDP), anonymized pharmacy identifier, and drug cost, can be retrieved from reMed [19, 20].

### Study design and outcome

A stratified cross-sectional design was used to fulfill the objectives. Before creating the strata, all prescriptions filled between 1 January 2015 and 23 May 2019 by privately insured patients registered in reMed were selected. We excluded filled prescriptions with invalid DIN or drug insurance number, drug cost or quantity equal to zero, inconsistency between cost and quantity dispensed, or patient age ≥ 65 years. Then, we formed strata of prescriptions with the same DIN, quantity dispensed, number of days supplied, pharmacy identifier, and edition of RAMQ’s List of Medications in force at the time of prescription dispensation to establish the ingredient list price. The RAMQ’s List of Medications sets out all prescription drugs covered by the PDP and their condition of coverage, including ingredient list price and is updated regularly to include new drugs and revised prices. A total of 38 updated editions of the RAMQ’s List of Medications were published over the study period. We created a unique number for each version of RAMQ’s List of Medications (i.e., 38 numbers) according to the period of time when the list was in force and we linked each prescription to the relevant list by using the date when the prescription was filled. Finally, each stratum of prescriptions filled by privately insured patients was matched with a stratum of prescriptions filled by patients insured by the PDP based on the same stratification factors. By matching on the number of the RAMQ’s List of Medications we controlled for the seniority of drugs by design. Our stratified design minimizes confounding in the quantification of the difference in drug cost between private drug plans and the PDP because the stratification factors are known to determine drug cost [1, 21–23].

The outcome was the drug cost, defined as the sum of the ingredient list price, wholesaler markup, and dispensing fee. As stated in the introduction, in Quebec the first two components of the drug cost are regulated by the RAMQ and are the same for patients covered by the PDP or by private drug plans, while dispensing fees may vary between drug plans.

### Statistical analyses

Descriptive analyses were used to summarize the characteristics of the study patients. The proportions of patients covered only by the PDP, only by a private drug plan, and who switched drug plan during the study period were calculated. Distributions of patients’ sex, age when the last prescription was filled, and year of enrollment were described using proportions. The mean and standard deviation (SD) of: the number of drug prescriptions filled, number of different molecules filled and number of pharmacies visited during the study period were calculated. Moreover, the total number of molecules and DINs in the sample, and the mean and standard deviation of the number of prescriptions per stratum were calculated.

To meet our objective, we estimated the mean drug cost separately for private drug plans and the PDP, using the strata as the unit of analysis. Then, we calculated two means: one mean of the means of drug cost for all private drug plan strata and one for all the PDP strata. This was done for all drugs in the sample. We then performed similar analyses for the subsets composed of: 1) all the strata containing the 10 most frequent drug classes in the sample, 2) all the strata containing the 10 most frequent molecules in the sample, and 3) all the strata containing the 10 most expensive molecules in the sample. In addition, we plotted the relationship between ingredient list price and absolute ($) and relative (%) differences in drug cost between private drug plans and the PDP. To calculate the relative difference in drug cost between private drug plans and PDP, we divided the difference in drug cost by the PDP drug cost because the latter is fixed and regulated by the PDP, and thus is used as a reference in our analysis. We also estimated the intra-stratum variation of drug cost with the standard deviation of prescriptions drug cost in a stratum and we calculated the proportion of strata with a standard deviation greater than zero.

Using linear regression models and the stratum as the unit of analysis, we estimated the mean differences in drug cost between private drug plans and the PDP, while taking into account the size of the strata. The dependent variable was the mean drug cost in the stratum and the independent variable was the type of drug insurance (private or PDP). The strata were weighted according to the number of prescriptions filled by privately insured patients they contained because it is considered as an important source of potential bias. Indeed, the prevalence of use of the drugs (i.e. volume of distribution) is likely to impact the dispensing fee (i.e. drugs with low volume might lead to higher dispensing fees) and consequently the cost of the drug. The use of the regression analysis allows to control for the size of the strata and consequently to control for the potential bias associated with the prevalence of use of the drugs in the estimation of the mean difference in drug cost, its standard deviation, and its 95% CI. By considering each filled prescription being independent of the other (i.e. not considering the strata via a regression analysis) we would have greatly under estimated the standard deviation of the mean difference in drug cost and provided smaller 95% CI, with the consequence of declaring differences statistically significant while they were not in reality.

To meet the secondary objective, we repeated the analyses separately for generic and innovator drugs for the 10 most frequent drug classes. Analyses were done with SAS software, version 9·4 (SAS Institute Inc., Cary, NC) and Excel (Microsoft, Redmond, WA).

## Results

There were 3 188 963 prescriptions filled by patients covered by private drug plans between 1 January 2015 and 23 May 2019 in reMed. After excluding prescriptions with invalid DIN or drug insurance number, drug cost or quantity equal to zero, or patient age ≥ 65 years, there were 2 335 846 prescriptions (Table 1). After excluding 203 additional prescriptions because of discrepancy between drug cost and quantity dispensed, we had 2 335 643 prescriptions and created 1 636 401 strata based on the DIN, quantity dispensed, number of days supplied, pharmacy identifier, and version of RAMQ’s List of Medications used to establish the cost of filled prescriptions. Of these private drug plan strata, 38 896 were one-to-one matched to strata of prescriptions filled under the PDP, for a total of 162 019 prescriptions and 77 792 strata. On average, each stratum was made of 4·2 (SD: 3·9) prescriptions. We analyzed a total of 363 molecules and 1 637 DINs. Table 2 presents an example of a stratum containing seven 30-day prescriptions of 30 tablets of Apo-Divalproex dispensed in one pharmacy and reimbursed by the PDP and a private drug plan. When considering only the 10 most frequent drug classes in the sample, the 10 most frequent molecules, and the 10 most expensive molecule subgroups, the numbers of matched strata were 29 998, 20 179, and 12 336, respectively. When generic and innovator drugs were analyzed separately for the 10 most frequent drug classes, there were 22 098 and 7 900 matched strata, respectively.

**Table 1:** Selection of the study sample

	**Private drug plan**	**PDP**
Filled prescriptions recorded in reMed between 1 January 2015 and 23 May 2019	3 188 963 prescriptions 38 064 patients	1 816 751 prescriptions 10 029 patients
Excluding filled prescriptions with drug cost or quantity equal to zero and invalid DIN or drug insurance number	2 629 317 prescriptions 33 441 patients	1 694 527 prescriptions 9 495 patients
Excluding prescriptions filled by patient 65 years old or more at the date of the last filled prescription	2 335 846 prescriptions 32 270 patients	558 273 prescriptions 6 244 patients
Excluding filled prescriptions inconsistency between cost and quantity dispensed	2 335 643 prescriptions 32 093 patients	557 675 prescriptions 6 187 patients
Strata of prescriptions based on DIN, quantity dispensed, number of days supplied, pharmacy identifier and RAMQ’s List of Medications	2 335 643 prescriptions 1 636 401 strata 32 093 patients	557 675 prescriptions 330 827 strata 6 187 patients
Matching strata of prescriptions filled by privately insured patients to strata of prescriptions filled by patients insured by the PDP (12 570 different patients in total)	100 809 prescriptions 38 896 strata 10 287patients	61 210 prescriptions 38 896 strata 3 599 patients

**Table 2:** Example of a stratum (stratum ID: 340)

Insurance type	DIN	Quantity dispensed	Number of days supplied	Pharmacy identifier	RAMQ’s List of Medications number	Cost ($)
Public	02239699	30	30	467269	13	12·54
Public	02239699	30	30	467269	13	12·54
Public	02239699	30	30	467269	13	12·54
Private	02239699	30	30	467269	13	14·89
Private	02239699	30	30	467269	13	14·89
Private	02239699	30	30	467269	13	14·89
Private	02239699	30	30	467269	13	14·89

The 162 019 prescriptions included in the sample were filled by 12 570 different patients. As shown in Table 3, most of the patients only had a private drug plan throughout the study period (*n* = 8 971). The distributions of sex, age, and year of enrolment were similar for patients covered by the PDP, those covered by private drug plans, and those who switched drug plans during the study period. The results suggest that patients covered by the PDP throughout the study and those who switched drug plans filled more prescriptions (17·5 and 26·5 vs 9·7) and used more molecules (2·6 and 3·7 vs 1·7) than patients privately insured throughout the study. The number of pharmacies visited was similar for all three groups of patients.

**Table 3:** Patients’ characteristics

	**Publicly insured patients**^a^	**Privately insured patients**^a^	**Privately and Publicly insured patients at different moments**^a^
**Characteristic**	**n (%)**	**n (%)**	**n (%)**
N	2 283 (100)	8 971 (100)	1 316 (100)
Sex			
Female	1 639 (71·8)	6 338 (70·6)	935 (71·1)
Male	644 (28·2)	2 633 (29·4)	381 (28·9)
Age (years) when patients filled their last prescription			
0–18	94 (4·1)	520 (5·8)	54 (4·1)
18–40	598 (26·2)	2 707 (30·2)	368 (28·0)
40–65	1 591 (69·7)	5 744 (64·0)	894 (67·0)
Year of enrolment in reMed			
2008–2014	1 717 (75·2)	7 325 (81·7)	1 075 (81·7)
2015–2019	566 (24·8)	1 646 (18·3)	241 (18·3)
Number of prescriptions filled per patient^a^ (mean (SD))	17·5 (32·5)	9·7 (16·4)	26·3 (38·7)
Number of different molecules filled per patient^a^ (mean (SD))	2·6 (2·2)	1·7 (1·2)	3·7 (2·6)
Number of pharmacies per patient^a^ (mean (SD))	1·3 (0·7)	1·2 (0·4)	1·5 (0·8)

*SD* standard deviation

^a^ During study period between 1 January 2015 and 23 May 2019

Table 4 presents the average drug cost for private drug plans and the PDP. We observed that the average drug cost was $62·34 for private drug plans and $52·99 for the PDP, with a crude difference of $9·35, representing a 17·6% greater cost for private drug plans. For generic drugs, we observed an average difference of $5·77 per prescription, or a 27·8% increase in cost in the private versus public drug plans. For innovator drugs, we observed an average difference of $19·61 per prescription, or a 15·1% higher cost in the private versus the public drug plans. We found that the intra-stratum standard deviation of the drug cost was higher than zero in 4 600 strata (11·8%) of private drug plans and in 4 349 strata (11·2%) of the PDP. Among strata from private drug plans with intra-stratum variations, the mean of the intra-stratum mean drug cost was $58·28 (SD: 273·43; Q1–Q3: 10·41–27·02) and the mean of the intra-stratum standard deviation of drug cost was $1·30 (SD: 4·09; Q1–Q3: 0·05–1·13), indicating very small variations in drug cost within strata. Among strata from the PDP with intra-stratum variations, corresponding figures were $48·01 (SD: 244·21; Q1–Q3: 10·15–20·29) and $0·42 (SD: 0·75; Q1–Q3: 0·27–0·42).

**Table 4:** Drug cost ($) according to drug insurance type

	**PDP**	**Private**		
	**Mean** (**SD)** **($)**	**Mean** (**SD)** **($)**	**Mean difference: private versus PDP ($)**	**Mean difference: private versus PDP (%)**
**Drug cost (number of strata)**				
All drugs (38 896)	52·99 (393·14)	62·34 (444·89)	9·35	17·6
Generic drugs (22 098)	20·78 (178·08)	26·55 (197·44)	5·77	27·8
Innovator drugs (7 900)	129·66 (563·84)	149·26 (656·60)	19·61	15·1

*SD* standard deviation

**Fig. 1:**
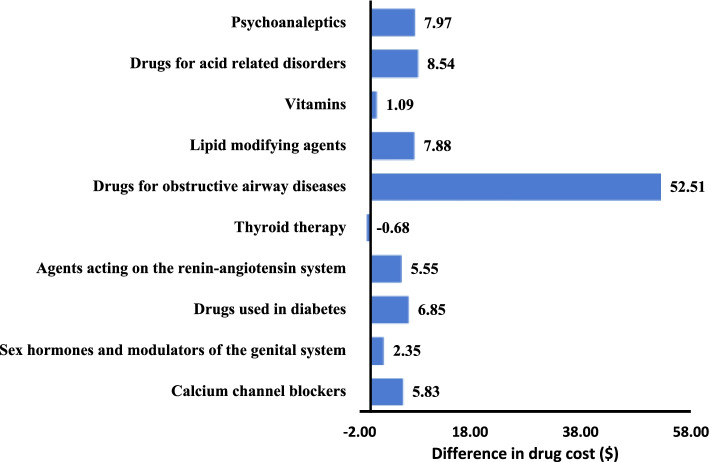
Difference in drug cost ($) private drug plans vs PDP for 10 most frequent drug classes (*n* = 29 998 strata)

**Fig. 2:**
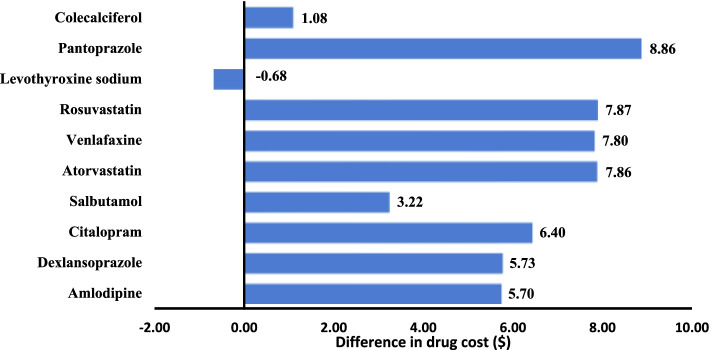
Difference in drug cost ($) private drug plans vs PDP for 10 most frequent molecules (*n* = 20 179 strata)

**Fig. 3:**
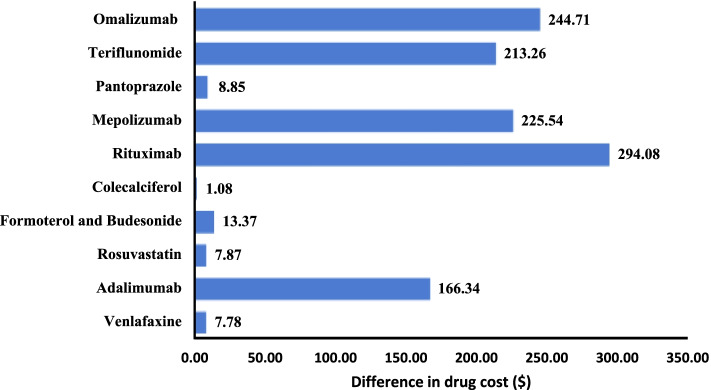
Difference in drug cost ($) private drug plans vs PDP for 10 most costly molecules (*n* = 12 336 strata)

Figures 1, 2 and 3 present the difference in mean drug cost between private drug plans and the PDP for selected drug subgroups. The mean drug cost for the 10 most frequent drug classes was higher for drugs reimbursed by private drug plans, except for thyroid therapy where the cost for patients covered by private drug plans was $0·68 (6·4%) lower (Fig. 1). We observed a similar trend for the 10 most frequent molecules (Fig. 2). The mean cost of the 10 most expensive molecules was higher for patients covered by a private drug plan (Fig. 3).

**Fig. 4:**
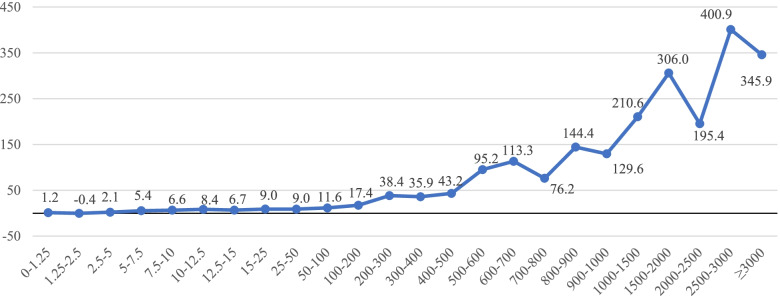
Difference in drug cost ($) private drug plans vs PDP according to RAMQ’s ingredient list price ($)

**Fig. 5:**
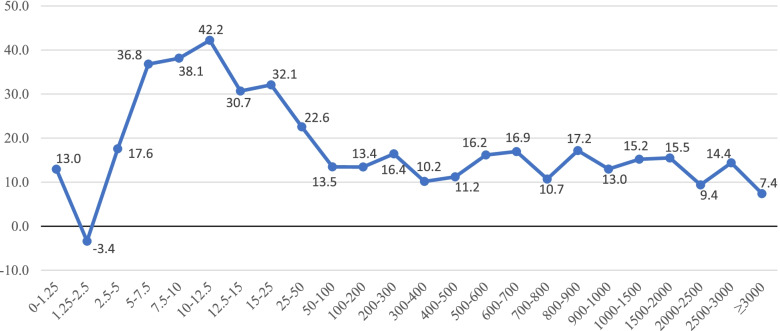
Relative difference in drug cost (%) private drug plans vs PDP according to RAMQ’s ingredient list price ($)

Figures 4 and 5 present the association between the difference in drug cost (in dollars and percentage, respectively) and the ingredient list price according to the RAMQ’s List of Medications. We observed a clear positive association between the ingredient list price and the mean difference in cost expressed in dollars, with patients covered by private drug plans paying more for their drugs. The largest relative difference in drug cost between private drug plans and the PDP was 42·2% for ingredient list prices ranging between $10·00 and $12·50.

The regression analysis revealed that being covered by a private drug plan compared with the PDP was associated with an average increase in drug cost of $9·35 (95% confidence interval [CI]: $5·68 to $13·01) (Table 5). In addition, the cost difference expressed in dollars was much higher for innovator drugs than generic drugs.

**Table 5:** Differences in drug cost ($) according to drug insurance type, as obtained from linear regression models

	**Private drug plans vs PDP**		
**Model (number of strata)**	**Mean difference ($)**		**95% CI**
All drugs (38 896)	9·35	5·68; 13·01	
Generic drugs (22 098)	5·77	3·74; 7·80	
Innovator drugs (7 900)	19·61	7·01; 32·20	

## Discussion

On average, drug cost was found to be 17·6% higher for drugs reimbursed by private insurance than by the PDP. The costs of innovator and generic drugs were 15·1% and 27·8% higher for private drug plans compared with the PDP, respectively. However, specific drug costs can sometimes be lower under a private drug plan, as in the case of levothyroxine sodium. On the other end, drug costs for prescriptions filled in a community pharmacy can be up to $400·00 higher for privately insured patients than patients insured by the PDP when the ingredient list price is between $2500·00 and $3000·00. In addition, our study showed that the difference in drug cost measured in dollars between private drug plans and the PDP increased with the ingredient list price. However, the relative difference measured in percentage did not show the same relationship, as it increased for ingredient list prices between $5·00 and $25·00, and decreased thereafter.

Our results were similar to those reported by previous studies conducted in Quebec. Indeed, a study by Desgagné showed that the costs of esomeprazole as an innovator and generic drug were 12·7% and 55·9% higher, respectively, for private drug plans compared with the PDP [12, 24]. Moreover, Levert showed that the costs of innovator and generic drugs were 17·0% and 37·0% higher, respectively, for private drug plans compared with the PDP in Quebec [13]. Studies conducted outside of Quebec reported drug costs up to 70% higher for privately insured patients than publicly insured patients but the comparison with our results is difficult since the regulation of the elements of the drug cost was either different from the one in place in Quebec or not clearly reported. Moreover, previous studies had various limitations, such as a small sample size, absence of statistical analyses, or lack of information about the study design, and the distribution of molecules differed between patients covered by private and public drug plans, giving rise to a risk of bias [10–18].

Our study has several strengths. First, we used a stratified design that minimized confounding bias in estimating the mean difference in drug cost between private drug plans and the PDP. Strata were defined according to all known and measurable factors that can influence the cost of a drug [1, 21–23]. We then used linear regression models to estimate the cost difference, while taking into account the size of each stratum. Furthermore, the sample size was large (162 019 prescriptions and 77 792 strata) and we analyzed 363 different molecules and 1 637 unique DINs. By limiting our study population to patients < 65 years, our study was representative of the population insured by private drug plans in Quebec.

However, the present results should be interpreted in light of the following limitations. reMed is not a random sample of patients covered by private drug plans in Quebec. The age and sex distributions are different from the population of Quebec. However, the participation in reMed is high (83%), participants are recruited in different rural and urban areas throughout the province, and the BMI, smoking status, and most used drug classes are similar between the Quebecers covered by a private drug plan and the patients in reMed [25–30]. We observed that in 11·5% of the strata, the intra-stratum standard deviation of the mean drug cost was higher than zero, meaning that drug cost varied between prescriptions of the stratum. This suggests that we could not control for all factors that affect drug cost within a stratum, although we controlled for the main cost drivers [1, 21–23]. This variability in cost within a stratum could be explained by data entry errors remaining despite the rigorous data quality control process in reMed. In addition, for privately insured patients, the dispensing fees can change over time. For publicly insured patients, the dispensing fees are determined yearly by government but it can change during a year based on the number of prescriptions served by the pharmacy. However, the intra-stratum variation for private drug plans and the PDP was found to be low compared with the mean drug cost, suggesting that it should not meaningfully affect the validity of this study.

The difference in mean drug cost observed in our study is driven by difference in dispensing fees between the PDP and private drug plans. Factors that can contribute to the higher dispensing fees in the private drug plans include a potential underfunding of the PDP through a fixed dispensing fee that did not keep pace with inflation, the need to support a large and increasingly expensive inventory of medications and the rising pharmacy operating expenses (rent, employee salaries and benefits, equipment, etc.), that challenges the profitability of the pharmacy.

## Conclusion

Our study shows that the drug cost is on average $9·35 (17·6%) higher for drugs reimbursed by private insurance than by the PDP, a gap driven by differences in dispensing fees between the two types of drug programs. These results inform patients, pharmacists, public health authorities and private insurers in Quebec and in Canada as well as any other decision makers managing a public drug plan about the size and the source of the drug cost differences between private insurance and PDP. They can support evidence-based decision making about drug insurance and pricing policies, as universal public drug programs and other mechanisms that aim at regulating dispensing fees and the total drug costs are debated in the political arena in several countries, including Canada. Our results could guide the development of drug plan policies and regulations that will ensure pharmacists' remuneration (including dispensing fees) reflect their skills, training, services and business expenses while decreasing inequities in the cost of drug distribution activities between the private and public sectors.

## Data Availability

The data that support the findings of this study are available from the reMed data extraction committee from the Université de Montréal but restrictions apply to the availability of these data, which were used under license for the current study, and so are not publicly available. Data are however available from Lucie Blais upon reasonable request and with permission of the reMed data extraction committee and the Human Scientific and Ethics Committee of the *Centre Intégré Universitaire de Santé et de Services Sociaux du Nord-de-l’Île-de-Montréal*.

## Abbreviations

PDP: Public Drug Plan
RAMQ: *Régie de l’Assurance Maladie du Québec*
reMed: *Registre de données sur les médicaments*
DIN: Drug identification number
